# Relative Roles of Grammar Knowledge and Vocabulary in the Reading Comprehension of EFL Elementary-School Learners: Direct, Mediating, and Form/Meaning-Distinct Effects

**DOI:** 10.3389/fpsyg.2022.827007

**Published:** 2022-06-21

**Authors:** Tsui-Chun Hu, Yao-Ting Sung, Hsing-Huang Liang, Tsung-Jen Chang, Yeh-Tai Chou

**Affiliations:** ^1^Transdisciplinary Program in College of Education, National Taiwan Normal University, Taipei, Taiwan; ^2^Department of Educational Psychology and Counseling, National Taiwan Normal University, Taipei, Taiwan; ^3^General Education Center, Asia Eastern University of Science and Technology, Taipei, Taiwan; ^4^Research Center for Psychological and Educational Testing, National Taiwan Normal University, Taipei, Taiwan

**Keywords:** grammar knowledge, reading comprehension, EFL, SEM, vocabulary

## Abstract

Despite the recognized importance of grammar knowledge to the reading comprehension of EFL learners, research findings on the relationships among grammar knowledge, vocabulary, and reading comprehension are inconclusive. Attention needs to be paid to issues such as the distinct roles of the two grammar knowledge components of form and meaning, and the direct and mediating roles of vocabulary in EFL reading comprehension. This study recruited 1,149 sixth graders as research participants to evaluate these issues. The measurement tools were standardized EFL competence tests for vocabulary size, grammar forms and meanings, and reading comprehension. Structural equation modeling (SEM) regression models indicated that vocabulary played a more-significant role in reading comprehension than grammar knowledge; moreover, the effects of grammar knowledge were reduced but still significant when grammar meanings were excluded. The SEM mediating model of this study also indicated that grammar knowledge not only exerted a direct effect on reading comprehension but also indirectly influenced reading comprehension *via* vocabulary.

## Introduction

The role of grammar knowledge in productive skills such as sentence patterns has been recognized in studies of speaking and writing (Basturkmen and Lewis, 2002; Droop and Verhoeven, 2003; Zhou, 2009). However, the literature suggests that the role of grammar knowledge in receptive skills, namely listening and reading, is equivocal and inconclusive (Buyl and Housen, 2015; Le Normand, 2018). Correlation-based and experimental studies have investigated the relationships between L2 vocabulary knowledge, L2 grammar knowledge, and L2 reading comprehension (Henning et al., 1981; Nassaji and Geva, 1999; Khaldieh, 2001; Van Gelderen et al., 2003, 2004; August, 2006; Shiotsu and Weir, 2007; Gottardo and Mueller, 2009; Guo and Roehrig, 2011; Morvay, 2012; Zhang, 2012; Nergis, 2013; Jeon and Yamashita, 2014; Kim and Cho, 2015; Lee, 2016; Susoy and Tanyer, 2019). However, these findings of these studies appear to be contradictory. Some studies determined that L2 grammar knowledge can have a strong impact on L2 reading comprehension (Henning et al., 1981; Van Gelderen et al., 2003; August, 2006; Shiotsu and Weir, 2007; Gottardo and Mueller, 2009; Guo and Roehrig, 2011; Nergis, 2013; Jeon and Yamashita, 2014; Kim and Cho, 2015), whereas other studies found that when the effects of vocabulary knowledge were partialed out, grammar knowledge played only a minor role in reading comprehension (Nassaji and Geva, 1999; Khaldieh, 2001; Van Gelderen et al., 2004; Morvay, 2012; Zhang, 2012; Lee, 2016; Susoy and Tanyer, 2019). Moreover, issues such as research methodologies (e.g., whether the influence of vocabulary was controlled), sample sizes, and the English proficiency of the subjects suggested that the research findings were both inconclusive and difficult to interpret. This indicates the necessity for further investigations to elucidate how L2 grammar knowledge affects L2 reading comprehension, and to further progress teaching and learning. The purpose of this study was to compare the relative contributions of grammar and vocabulary knowledge to EFL reading comprehension.

Grammar knowledge includes the understanding of subject–verb agreement, tenses, articles, and word order (Qian, 2002). Different terminologies of grammar knowledge are used in the literature, with common expressions such as grammar knowledge, morphosyntactic, syntactic awareness, syntactic knowledge, and syntactic parsing being popular in research on reading (Tunmer et al., 1987; Gaux and Gombert, 1999; Morvay, 2012; Zhang, 2012; Jeon and Yamashita, 2014; Fang et al., 2017; Susoy and Tanyer, 2019). Few studies have explored the role of grammar knowledge in L2 reading comprehension (Urquhart and Weir, 1998; Shiotsu and Weir, 2007; Jung, 2009; reviewed by Choi and Zhang, 2021). This may be because the communicative language teaching (CLT) approach (Savignon, 2008; Chung and Huang, 2009) has been widely used in classrooms since the mid-1970s, which focuses on fluency in language learning, but not accuracy. The role of grammar instruction in CLT is therefore a controversial issue (Wong and Barrea-Marlys, 2012). August (2006) studied the roles of L1 and L2 grammar knowledge in L1 and L2 reading comprehension among Spanish-speaking college students. Their correlation and regression analysis indicated that L2 grammar was correlated with L2 reading comprehension (*r* = 0.031). Similarly, Gottardo and Mueller (2009) conducted a longitudinal study of the roles of language factors in predicting reading comprehension among first- and second-grade Spanish students learning to read English and found that L2 grammar knowledge was significantly correlated with English reading comprehension (*r* = 0.40). Four other studies indicated that L2 grammar had an important role in L2 reading comprehension among EFL learners (Henning et al., 1981; Van Gelderen et al., 2003; Guo and Roehrig, 2011; Nergis, 2013). A study by Henning et al. (1981) of Egyptian EFL learners in secondary education reinforced the findings of the above-mentioned studies, with their results indicating that L2 grammar knowledge had a strong correlation with L2 reading comprehension (*r* = 0.65). The findings of Nergis (2013) for college-level EFL learners in Istanbul were similar, with syntactic awareness having a stronger correlation with L2 reading comprehension (*r* = 0.625) than with vocabulary depth knowledge (*r* = 0.533). Moreover, their multiple regression analysis indicated that syntactic awareness was a strong predictor of L2 reading comprehension. Some researchers have used structural equation modeling (SEM) to analyze data (Van Gelderen et al., 2003; Guo and Roehrig, 2011). Van Gelderen et al. (2003) found a substantial relationship between the L2 grammar knowledge and L2 reading comprehension of on adolescent Dutch EFL learners (*β =* 0.51). Moreover, their SEM analysis indicated that L2 grammar knowledge played a significant role in L2 reading comprehension when controlling for metacognitive knowledge and processing.

With a focus on Chinese EFL college students, Guo and Roehrig (2011) investigated the roles of metacognitive awareness in reading strategies and linguistic knowledge in L2 reading comprehension. Their results indicated that L2 language knowledge (about both vocabulary and grammar) was a strong predictor of L2 reading comprehension, since vocabulary and grammar knowledge did not independently explain reading comprehension. Jeon and Yamashita (2014) constructed a meta-analysis to determine if L2 grammar knowledge was a strong predictor of L2 reading comprehension, and in contrast found that grammar knowledge and reading comprehension were strongly correlated (*r* = 0.85), with this correlation being stronger than that between vocabulary knowledge and reading comprehension. Notably, despite the findings of a moderate-to-strong correlation between grammar knowledge and reading comprehension, the relationship between grammar knowledge and reading comprehension must be further examined after considering the possible influences of vocabulary knowledge, which were not appropriately controlled in those studies.

Reading has been regarded as a receptive language skill for understanding the words, sentences, and meaning in texts (Jung, 2009). Studies have consistently found that L2 vocabulary plays a significant role in L2 reading comprehension (Nassaji and Geva, 1999; Khaldieh, 2001; Van Gelderen et al., 2004; Morvay, 2012; Zhang, 2012; Kim and Cho, 2015; Lee, 2016; Susoy and Tanyer, 2019). L2 vocabulary knowledge strongly contributes to L2 reading comprehension, with a ranging from 0.54 to 0.90 (Nassaji and Geva, 1999; Khaldieh, 2001; Morvay, 2012; Susoy and Tanyer, 2019).

The primary purpose of the study by Kim and Cho (2015) was to determine the respective roles of vocabulary and grammar knowledge in reading comprehension among Korean EFL learners with high, intermediate, and low proficiency levels. Their multiple regression results indicated a significant correlation between L2 vocabulary knowledge and L2 reading comprehension (*r* [106] = 0.319). Those authors also reported that L2 grammar knowledge was a significant positive predictor of L2 reading comprehension when controlling for vocabulary in the high-proficiency group; however, the effect of grammar knowledge was not significant after controlling for vocabulary in the intermediate-proficiency group. Van Gelderen et al. (2004), Shiotsu and Weir (2007), and Zhang (2012) used SEM to determine the effects of grammar knowledge or vocabulary on EFL reading comprehension after partialing out the influences of both, which produced inconsistent findings. For example, Shiotsu and Weir (2007) focused on Japanese EFL college learners, and found L2 grammar knowledge to be a strong predictor of L2 reading comprehension when controlling for the effect of vocabulary knowledge. Zhang (2012) investigated the relative contribution of linguistic knowledge to L2 reading comprehension among Chinese-speaking advanced EFL learners, and in contrast found that L2 grammar knowledge did not significantly affect L2 reading comprehension when controlling for vocabulary effects (*β* = 0.660). Similarly, Van Gelderen et al. (2004) investigated the relative influences of linguistic knowledge, processing speed, and metacognitive knowledge on L1 and L2 reading comprehension among Dutch EFL learners in secondary education, and reported that L2 grammar knowledge did not significantly contribute to L2 reading comprehension when controlling for the effect of vocabulary knowledge. The above-mentioned studies suggest that the role of vocabulary in the relationship between grammar knowledge and reading comprehension remains inconclusive.

The above-mentioned researchers used various methods to investigate the relationship between L2 grammar and L2 reading comprehension, but some aspects of their studies led to the results being inconsistent and difficult to interpret. The first limitation was that the number of participants recruited for investigating the relative contribution of grammar knowledge and vocabulary on EFL comprehension should be increased to increase the validity of the evidence. Previous studies mostly recruited high-school and college students, and adult learners who had received explicit/direct EFL grammar lessons for a certain period (Henning et al., 1981; Nassaji and Geva, 1999; Khaldieh, 2001; Van Gelderen et al., 2003, 2004; August, 2006; Shiotsu and Weir, 2007; Guo and Roehrig, 2011; Morvay, 2012; Zhang, 2012; Kim and Cho, 2015; Lee, 2016; Susoy and Tanyer, 2019). Literature on EFL elementary-school learners who received shorter periods of English grammar instruction (relative to their high school and college counterparts) is scarce, which may be disadvantageous for learning the forms or patterns of the English language. Recruiting learners at an elementary-school level with low exposure to explicit EFL grammar instruction would yield evidence on the relationship between L2 grammar knowledge and L2 reading comprehension. Furthermore, several previous studies applied SEM (Van Gelderen et al., 2003, 2004; Shiotsu and Weir, 2007; Gottardo and Mueller, 2009; Guo and Roehrig, 2011; Zhang, 2012) to samples smaller than 300, which Comrey and Lee (1992) considered to be the minimum number proposed for SEM data analyses. More-diverse learning levels and more participants would produce more valid evidence about the relative contribution of grammar knowledge to EFL reading comprehension.

The second issue was that the interplay of the two variables was not addressed in addition to comparing the relative significance of grammar knowledge and vocabulary. Previous studies employed SEM to determine the direct effects (*β* values) of grammar knowledge and vocabulary on EFL reading comprehension, compared the two relative effects, and postulated which of these independent variables had the greatest effect on the dependent variable. The cognitive-component analysis (Carr and Levy, 1990) of EFL reading comprehensions an approach that may help to define the roles of different cognitive components in EFL reading. However, for an interactive approach to reading (Grabe, 1991), only focus on the comparisons of the independent contributions of the two components might neglect the interaction effects between grammar knowledge, vocabulary, and EFL reading comprehension. For example, previous research (e.g., Shiotsu and Weir, 2007; Kim and Cho, 2015) found moderate-to-strong correlations between grammar knowledge and vocabulary. Furthermore, previous research postulated that reading context, such as morphology cues, syntax collocations, synonyms, and background knowledge influence inferences of word meanings and reading comprehension for L1 and L2 learners (Laufer and Bensoussan, 1982; Chern, 1993; Engelbart and Theuerkauf, 1999; Walters, 2004; Zhang and Koda, 2012). The close relationships among the three variables warrant further questions to be addressed: (a) in addition to the direct effects, does vocabulary have a mediating effect on the relationship between grammar knowledge and EFL reading comprehension? and (b) does grammar knowledge both directly influence EFL reading comprehension and indirectly exert its influence through vocabulary?

The third issue was that the distinct function of the two important components of grammar knowledge, grammar form and grammar meaning, needed to be evaluated after addressing the possible confounding variables between reading comprehension and grammar knowledge. Previous researchers have proposed that the semantic/meaning component is an element of grammar knowledge, and also the main component of reading comprehension. For example, Larsen-Freeman (2001) proposed a framework of three-dimensional grammar comprising the components of form/structure, meaning/semantic, and use/pragmatics, which are often integrated into grammar knowledge learning. For grammatical ability assessments, Purpura (2004, p. 89) defined grammatical ability as involving “the capacity to realize grammatical knowledge accurately and meaningfully in test-taking or other language-use contexts,” proposing that the accuracy of use (form) and the understanding of context for use (meaning) are closely related in grammatical learning. Previous researchers (Alderson, 1993; Urquhart and Weir, 1998) also proposed that the overlapped content of the testing instruments should be minimized when evaluating the relationship between syntax and reading comprehension; that is, researchers should make their instruments “pure” so as to independently reflect the construct of each instrument. This postulation is not easy to realize. Shiotsu and Weir (2007) attempted to control the distinct components of their syntax and reading comprehension measurement tools, but found that the relationship between EFL grammar knowledge and reading comprehension remained strong (*r* = 0.85 and 0.62 in their second and third analyses, respectively). The strong correlation between grammar knowledge and reading comprehension may be a confounding factor that influences the direct and mediating effects of grammar knowledge on reading comprehension. Comparing the results of grammar knowledge tests between those that include a meaning/context component and those that do not will therefore be helpful for determining the direct and mediating effects of grammar knowledge on reading comprehension.

The purpose of the current study was to overcome the limitations of the above-mentioned studies. First, a large sample (*N* = 1,149) of learners at the elementary-school level with restricted experience of EFL grammar instruction was recruited as the research participants, which may help to provide more valid evidence for the relationships between grammar knowledge, vocabulary, and reading comprehension. Second, research investigating both the direct and mediating effects of grammar knowledge on reading comprehension is scarce, and so regression and mediation models in SEM were constructed in this study. The former models focused on the relative contribution of grammar knowledge and vocabulary to reading comprehension, while the latter models examined the possible interplay of grammar knowledge and vocabulary, about whether grammar knowledge influences reading comprehension directly or indirectly *via* mediation from the vocabulary. Third, this study attempted to improve the distinction between grammar form (GF) and grammar meaning (GM) in grammar knowledge tests and compared the direct and mediating effects under the conditions of test scores with GF scores only and with both GF and GM scores.

## Materials and Methods

### Participants

The participants in this study were 1,149 EFL sixth graders at elementary schools in Taipei City and New Taipei City. They comprised 661 (57.53%) males and 488 (42.47%) females aged 12 or 13 years. The participants had received communicative language teaching (Huang, 2016) for 3 years. During that period, sentence patterns of the English language were embedded in speaking, listening, reading, and writing activities instead of direct instructions of grammar knowledge. Each participant attended three 40-min English classes each week. None of the 1,149 students had accepted the administration of the DCEC tests described below for the purposes of evaluating their psychometrical properties.

### Instruments

The Diagnosis and Certification of English Competence (DCEC) test is based on the English national curriculum guidelines of Taiwan and developed by the Research Center for Psychological and Educational Testing at National Taiwan Normal University. The DCEC test is used to assess the four English language skills of vocabulary size (DCEC-VS), grammar (DCEC-G), listening, and reading comprehension (DCEC-RC), and it provides detailed information and learning suggestions for the learning performance of examinees (Hu et al., 2020; Hu and Hsu, 2020).

The DCEC test divides language proficiency into five levels (D1 to D5) and was designed to assess the English proficiency of EFL examinees from grades 3 to 9, which were identical to the grade-level proficiencies. The DCEC test was implemented using computerized adaptive testing (CAT) techniques, which decreases the time requirements and improves the efficiency of the test.

#### Diagnosis and Certification of English Competence-Vocabulary Size

The DCEC-VS test was designed to estimate the vocabulary size of learners using 2,000 words listed in the curriculum guidelines of grades 3–9 to represent their ability in recognizing the meaning of a word (Table 1). In order to obtain the item-difficulty parameters, 3,500 participants (325, 381, 554, 1,251, and 1,790 from grades 3-4, 5-6, 7, 8, and 9, respectively) completed the test. Item response theory (a two-parameter logistic model; Embretson and Reise, 2000) was adopted to estimate the vocabulary competence of the participants and determine the words that had and had not been mastered by the participants. Based on the two-parameter logistic model, the DCEC-VS estimated students’ vocabulary competence (θ, ranging from −3 to 3), which was then transformed into scores ranging from 0 to 250. Scores were considered to be more comprehensible for students.

**Table 1:** Vocabulary size according to DCEC level.

DCEC level	Vocabulary size used in DCEC-VS	Grand total
DCEC 1	125	125
DCEC 2	156	281
DCEC 3	273	554
DCEC 4	697	1,251
DCEC 5	839	2,090

The evaluation of the DCEC-VS test involved assessments of reliability, criterion-related validity, and construct validity. The participants were 960 elementary- and middle-school students. First, the group-level conditional reliability coefficients (Raju et al., 2007) for grades 3–4, 5–6, 7, 8, and 9 were 0.95, 0.93, 0.93, 0.90, and 0.89, respectively. Second, for criterion-related validity, the DCEC-VS scores of examinees were compared with their English language performance in schools (represented by *z* scores); the correlation coefficients for grades 3-4, 5-6, 7, 8, and 9 were 0.61, 0.74, 0.52, 0.66, and 0.62, respectively. Moreover, the DCEC-VS and reading scores of ninth-grade participants were compared based on their Basic Competence Test (now called the Comprehensive Assessment Program, CAP), which is designed for the ninth graders and serves as the entrance examination for the admission to senior high schools in Taiwan (Sung et al., 2016), and had a correlation coefficient of 0.67. Third, construct validities indicated that participants at each DCEC level had significantly different vocabulary sizes (183, 334, 617, 825, and 1,264 for grades 3-4, 5-6, 7, 8, and 9, respectively). The analysis of the variance of the participant vocabulary sizes at different DCEC levels revealed significant differences in vocabulary sizes between the five DCEC levels (*F*_4,856_ = 1,550, *p* < 0.001). Additionally, *post-hoc* analyses were performed using Tukey’s HSD (Tukey’s honest significant difference), which indicated significant differences in every pairwise comparison between each level. These results therefore indicated that DCEC-VS levels were correlated with the vocabulary size of EFL learners.

#### Diagnosis and Certification of English Competence-Grammer

The DCEC-G test is a grammar test based on the national curriculum guidelines of Taiwan and the English textbooks commonly used in mainstream local schools. The DCEC-G test measures two components of grammar knowledge: GF and GM (Alderson, 1993; Larsen-Freeman, 2001; Purpura, 2004). GF focuses on English sentence structure and grammar items, while GM focuses on knowledge of grammar structure and learner capability when they are applying internalized rules for communicating within language contexts (Ellis, 1997; Urquhart and Weir, 1998; Shiotsu and Weir, 2007). To cater to the English levels of students with different grades in Taiwan, the DCEC-G test types include unscrambling sentences, multiple-choice questions, and picture-based questions.

**Figure 1:**
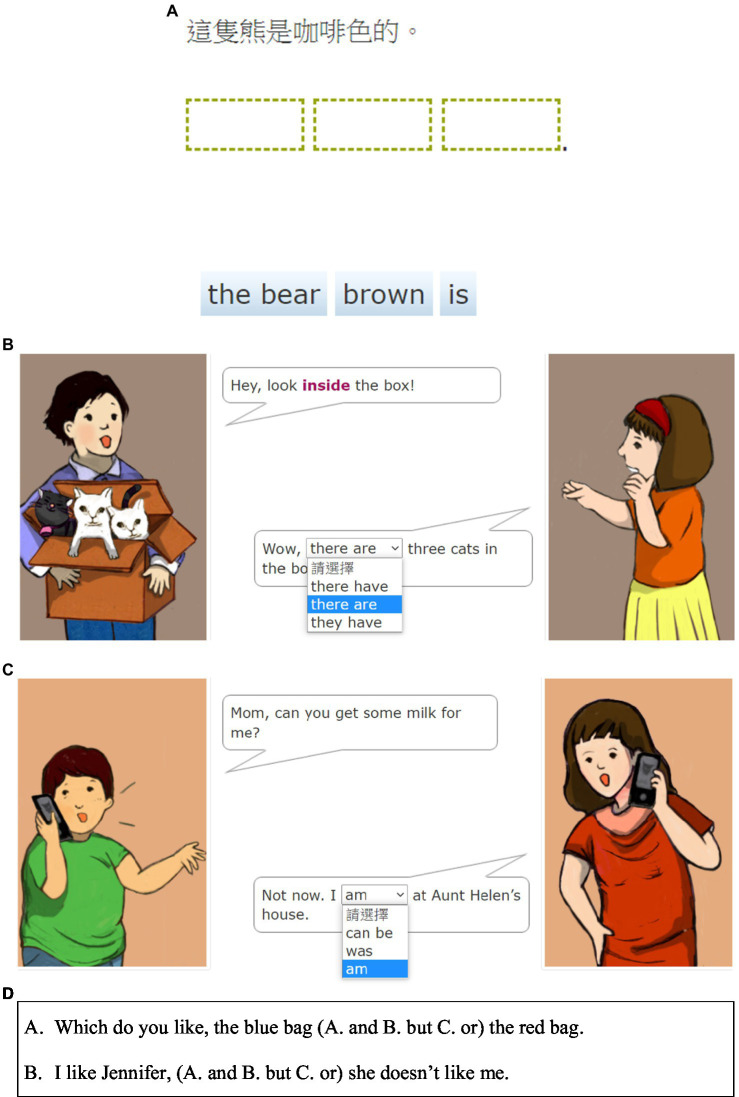
**(A)** An example of a GF question. **(B)** An example of a GF question. **(C)** An example of a GM question. **(D)** An example of a GM question.

Figure 1A shows a GF question mostly focused on testing the sentence structure knowledge of the students. Given the meaning when written in Chinese, students were asked to unscramble the order of three different parts (“the bear,” “brown,” and “is”) with the correct answer being “The bear is brown.” This type of question tends to minimize the interference from other elements (e.g., the reading comprehension ability of students) and mostly focuses on its grammar/sentence pattern elements; in this case, the “subject–verb–adjective” sentence pattern. Figure 1B shows another GF question with a multiple-choice format. Although the dialogue context is provided here, the ability of students to understand the context is actually irrelevant to them choosing the correct answer. Instead, they only need to know which choice helps them to construct a grammatically correct sentence (i.e., “There are three cats in the box”), because the question focuses on grammatical form but not meaning. In contrast, context is always provided for items that test how students understand meaning and usage of a certain sentence pattern (GM questions), which was provided by pictures with additional clues. Students were asked to determine and choose the answer that best suits the situation in the corresponding context, thereby testing the ability of students to apply a grammatical pattern they have learnt to different contexts. For example, in Figure 1C, all three choices can construct a grammatically correct sentence: “I can be at Aunt Helen’s house.”/“I was at Aunt Helen’s house.”/“I am at Aunt Helen’s house.” Whether a student can answer this question correctly depends on his/her ability to understand the given context and choose a suitable answer. Students need to know to put a “be-verb”after the subject “I” here, and since the context was an ongoing conversation, they must use the present tense option “am” to fit this situation. This suggests that students learn grammatical items from this question. They must also understand the entire situation in the context to apply the learnt grammatical item. Figure 1D shows another type of GM test item that did not involve a conversation as a context, but instead comprised a blank-filling sentence with three choices denoting different meanings. Students must understand the meaning of each choice to choose the correct answer. This test item tested the conjunction word knowledge of the students, with the options “and,” “but,” and “or” all denoting different relationships between the two clauses of each sentence. Students must understand these differences to answer the question correctly. Therefore, while students were learning the grammar item “conjunction words,” they also needed to know the meaning of such words and apply them in various contexts.

There were seven grammar concepts: word order, tense and aspect, auxiliary verbs, special sentence structure, agreement, wh-questions, and miscellaneous. In each grammar category, it is further divided into various sentence patterns, each containing 1 GF test item and 2 or 3 GM test items. The total number of sentence patterns covered for grades 3–9 was 111, and the total number of test items was 316.

The DCEC-G test was designed using CAT, and the item response theory (two-parameter logistic model; Embretson and Reise, 2000) was adopted for the multiple-choice tests to estimate the grammatical competence of participants. For the questions involving unscrambling a word order, each correctly filled blank of each item was awarded 1 point, and the partial credit model (Masters, 1982) was used for the test data analysis. Based on the partial credit model, the DCEC-G estimated students’ grammatical competence (θ, ranging from −3 to 3), which was then transformed into scores ranging from 0 to 250 for students’ easier access to the meaning of the scores. The scores of the meaning subskill and form subskill of the DECE-G were also calculated in the same way, and the score of each subskill ranged from 0 to100. The evaluation study included 4,268 participants (348, 1,082, 1,015, 826, and 1,055 participants in grades 3-4, 5-6, 7, 8, and 9, respectively) recruited from elementary and middle schools. A criterion-related validity study was conducted to compare the DCEC-G scores and English grades in schools (represented by *z* scores) of the participants, which yielded correlation coefficients for grades 3-4, 5-6, 7, 8, and 9 of 0.81, 0.76, 0.84, 0.77, and 0.59, respectively.

#### Diagnosis and Certification of English Competence-Reading Comprehension

The DCEC-RC test is a computerized adaptive test aligned to the curriculum guidelines in Taiwan. Test items for levels D1 and D2 assess the primary reading comprehension skills of beginner EFL learners, including extraction, integration, and inferencing. Test items for levels D3 to D5 mostly focus on local and global inferences for diverse genres such as text messages, letters, and interpreting tables and charts from reading materials (Supplementary Appendix A). In order to meet the learning conditions, texts in the DCEC-RC tests are based on vocabulary size (as listed in Table 1). There are 72, 76, 75, 72 and 87 items for 3–4, 5–6, 7, 8, and 9 grades, respectively. The psychometric properties of the DCEC-RC test consist of both classic test theory and item response theory (IRT) approaches. For the traditional item and reliability analyses, 725 participants (348, 110, 110, 87, and 70 in grades 3–4, 5–6, 7, 8, and 9, respectively) were recruited from elementary and middle schools. Cronbach’s α coefficients for internal consistency and reliability in grades 3–4, 5–6, 7, 8, and 9 ranged from 0.73 to 0.94. Another sample of 1,870 participants (323, 342, 326, 477, 402 for grades 3–4, 5–6, 7, 8, and 9, respectively) were recruited for the IRT-based parameter estimation. Since the DCEC-RC test consists of two or three reading comprehension dimensions, we adopted the multidimensional random coefficients multinomial logit model (Adams et al., 1997) for the data analysis. All the 382 items were then pooled to form an item bank with the parameters of item difficulties estimated by the IRT model. Based on the reading comprehension ability (θ, ranging from −3 to 3) estimated by the IRT model, the DECE-RC transformed the θ into scores ranging from 0 to 250, which were easier for students to comprehend. The scores of the subskills of the DCEC-RC (i.e., the extraction, integration, and inference competence) were calculated in the same way, and the score of each subskill ranged from 0 to 100. Regarding the validity of the DCEC test, the correlations between DCEC-RC scores and English school grades (represented by *z* scores) of the first sample (725 participants) were assessed, which yielded correlation coefficients for grades 3–4, 5–6, 7, 8, and 9 of 0.51, 0.50, 0.78, 0.74, and 0.71, respectively. Moreover, the DCEC-RC scores of 412 ninth graders were compared with their reading scores from the Comprehensive Assessment Program (Sung et al., 2016), which produced a correlation coefficient of 0.79. These results provided strong evidence that DCEC-RC scores can be used to estimate the English performance of EFL learners in early stages.

### Procedure

Data were collected from June 2016 to October 2017. We adopted a counterbalancing approach when administering the DCEC-VS, DCEC-G, and DCEC-RC tests. Participants had 10 min of rest after each test, and required around 90 min to complete each test. The parents or guardians of all participants signed an informed-consent form.

### Data Analysis

Data were analyzed using the Mplus 8 and SPSS 23.0 programs. The descriptive data, correlation matrix, and mean distributions were examined first. The mean scores and skewness and kurtosis values for each subskill of grammar knowledge and EFL reading comprehension, and the overall scores were obtained for the DCEC-VS, DCEC-G, and DCEC-RC tests. Chou and Bentler (1995) suggested that normally distributed data have skew and kurtosis values between −2 and 2, and based on this the scores did not significantly deviate from the normal distribution. We calculated descriptive statistics, including means, standard deviations, and skewness and kurtosis values. Pearson’s correlation coefficients were calculated to identify significant relationships among the variables.

**Figure 2:**
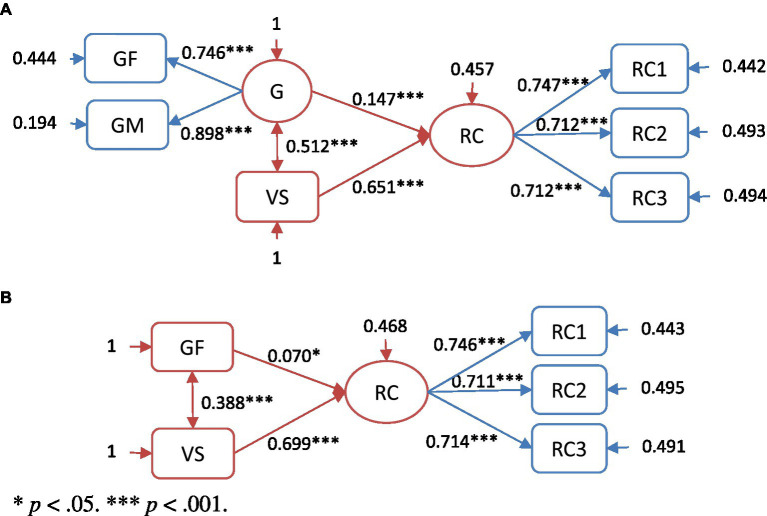
**(A)** The basic regression model. **(B)** The GF regression model. ^*^p < 0.05, ^***^p < 0.001.

**Figure 3:**
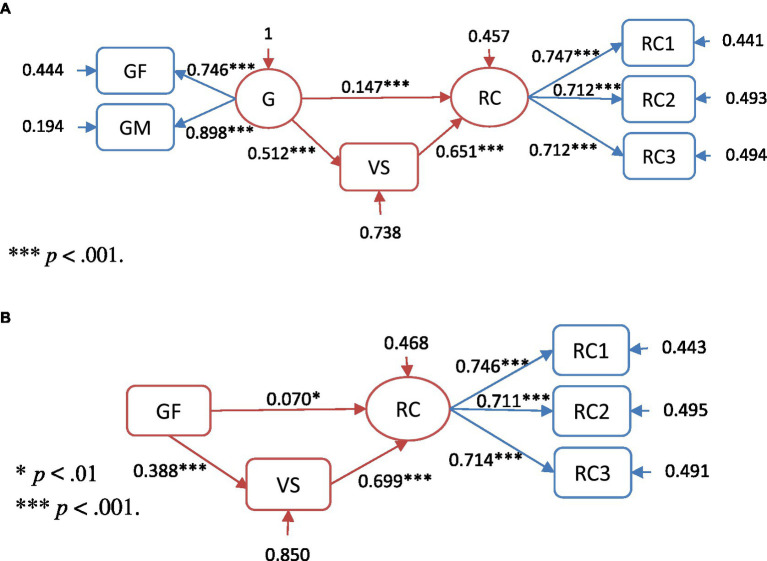
**(A)** Visualization of the structural model with standardized direct effects. ^***^p < 0.001. **(B)** Visualization of the structural model with the standardized direct effects of the GF model. ^*^p < 0.01 and ^***^p < 0.001.

Regarding the direct and mediating effects of grammar knowledge and vocabulary on reading comprehension, SEM analysis was performed on both the basic regression (Figures 2A,B) and mediation (Figures 3A,B) models. The measurement and the structural models were tested using the maximum likelihood estimation method. Since χ^2^ values are sensitive to sample size, fit indices were adopted in the covariance structure analysis to determine the goodness of fit to the model. Hu and Bentler (1999) suggested that CFI and TLI values ≥ 0.95 and RMSEA ≤ 0.06 indicate a very good model fit. To determine whether vocabulary mediated the influence of grammar knowledge on reading comprehension, Mplus 8 was used for mediation analysis. Indirect effects were tested using bias-corrected bootstrapping (*N* = 1,000) and 95% confidence intervals (CIs) for the indices. A parameter was considered significant when its 95% bootstrapped CI did not include zero.

## Results

### Descriptive Statistics

Table 2 lists the descriptive data of the six measures used in this study. Pearson’s correlation coefficients for all measures are listed in Table 3. All observed variables had significant and positive correlations. DCEC–*VS* scores were significantly correlated with DCEC–G scores (*r* = 0.82, *p* < 0.001), DCEC–RC scores (*r* = 0.61, *p* < 0.001), and the scores for each subskill. According to the rules of thumb of Cohen (1988), correlations with coefficients of 0.30–0.50 and >0.50 are considered to be moderate and strong, respectively. The correlations between DCEC–VS, DCEC–G, and DCEC–RC scores were therefore strong. It is notable that when the form (GF) and meaning (GM) of grammar knowledge were separated, the correlation coefficients between grammar knowledge and reading comprehension were significantly reduced. GF was significantly correlated with the extraction (*r* = 0.26, *p* < 0.001), integration (*r* = 0.24, *p* < 0.001), and inferences (*r* = 0.24, *p* < 0.001) of the DCEC–RC test, as was GM (*r* = 0.33, *p* < 0.001; *r* = 0.33, *p* < 0.001; and *r* = 0.29, *p* < 0.001, respectively).

**Table 2:** Descriptive statistics for DCEC–*VS*, DCEC–G, and DCEC–RC subtests and subtest subskills (*N* = 1,149).

	Mean	SD	Skewness	Kurtosis	Min.	Max.
DCEC-VS	58.38	58.27	1.35	1.00	1	233
DCEC-G	76.52	53.61			7	243
Form	69.18	12.64	−0.81	1.48	11	100
Meaning	73.01	15.29	−0.91	0.33	26	100
DCEC-RC	37.03	34.32			7	248
Extraction	51.39	23.60	0.15	−0.73	0	100
Integration	43.44	23.58	0.38	−0.59	0	100
Inferences	47.53	22.13	0.23	−0.78	0	100

**Table 3:** Correlations among all examined variables (*N* = 1,149).

Variable	1	2	3	4	5	6	7
1. DCEC-VS	—						
2. DCEC-G	0.82	—					
3. Form	0.39	0.38	—				
4. Meaning	0.46	0.50	0.67	—			
5. DCEC-RC	0.61	0.55	0.24	0.27	—		
6. Extraction	0.52	0.49	0.26	0.33	0.41	—	
7. Integration	0.54	0.55	0.24	0.33	0.38	0.54	—
8. Inferences	0.52	0.50	0.24	0.29	0.44	0.55	0.48

*p <* *0.001*.

### Regression Model Testing

The testing of the basic regression model (Figure 2A) revealed χ^2^ (7, *N* = 1,149) = 14.88, *p* < 0.05; CFI = 0.997; TLI = 0.993; and RMSEA = 0.031, 95% CI = 0.007–0.054 (Table 4). Based on the cutoff values suggested by Hu and Bentler (1999), the model had a very good fit. The standardized regression weights (*β*) of the basic regression model provide insight into the relative contributions of its components to explain EFL reading comprehension. It was initially found that 54% of EFL reading comprehension can be explained by the components combined. Our dependent variables appeared to be explained by their components. Vocabulary made a significant contribution to EFL reading comprehension (*β* = 0.651, *p* < 0.001), but grammar knowledge also had a significant regression weight (*β* = 0.147, *p* < 0.001).

**Table 4:** Model fit indices for the basic regression and form regression models.

Model	χ^2^	Df	RMSEA	CFI	TLI	SRMR
Basic regression	14.877	7	0.031	0.997	0.993	0.012
Form regression	9.881	4	0.036	0.996	0.991	0.011

To avoid the GM subskill interfering with grammar knowledge, the GF regression model (Figure 2B) only included the form subskill of GF. The testing of the GF regression model revealed χ^2^ (4, *N* = 1,149) = 9.881, *p* < 0.05; CFI = 0.996; TLI = 0.991; and RMSEA = 0.036, 95% CI = 0.006–0.065, indicating a very good fit (Table 4). Vocabulary made a significant contribution to the GF regression model (*β* = 0.699, *p* < 0.001), but GF also had a significant regression weight (*β* = 0.070, *p* < 0.05). These two components explained about 53.2% of the variance within EFL reading comprehension.

### Mediation Model Testing

The mediation models, as shown in Figures 3A,B, postulated that EFL reading comprehension was predicted by two variables: vocabulary and the latent variable of grammar knowledge. In addition, vocabulary was also predicted by grammar knowledge. When testing the structural model, the factor loading of grammar knowledge was fixed at 1.0. A mediation analysis was conducted using Mplus 8 to determine if vocabulary mediated the influence of grammar knowledge on EFL reading comprehension.

The fit statistics of mediation model were as follows: *χ*^2^ (7, *N* = 1,149) = 14.877, *p* < 0.05; *CFI* = 0.997; *TLI* = 0.993; and *RMSEA* = 0.031, 95% *CI* = 0.007–0.054, indicating an excellent fit. The mediation model (Figure 3A) evaluated the strength of the indirect relationship while controlling for the direct effect of grammar knowledge on EFL reading comprehension. In this model, vocabulary was indicated to mediate the relationship between grammar knowledge and EFL reading comprehension.

As shown in Figure 3A, grammar knowledge significantly predicted the vocabulary mediator (*β* = 0.512, *p* < 0.001), and the vocabulary mediator significantly predicted EFL reading comprehension (*β* = 0.651, *p* < 0.001). Grammar knowledge and vocabulary together explained about 54.3% of the variance within EFL reading comprehension. As listed in Table 5, the baseline structural model suggested a significant indirect effect of grammar knowledge on EFL reading comprehension through vocabulary (*β* = 0.333, *p* < 0.001). Both the direct and indirect effects were significant, and the total effects of grammar knowledge on EFL reading comprehension were significant (*β* = 0.480, *p* < 0.001), indicating that those with better grammar knowledge had better EFL reading comprehension. The direct effect of grammar knowledge predicted EFL reading comprehension after controlling for vocabulary, which was also significant and smaller than the total effect (*β* = 0.147, *p* < 0.001). However, the bootstrapped 95% CIs for both the total (0.431–0.530) and direct (0.086–0.208) effect models did not include zero, suggesting that the effect of grammar knowledge on EFL reading comprehension was only a partial mediator.

**Table 5:** Mediation effect of vocabulary.

	*β*	SE	Value of *p*
Grammar knowledge ➔ EFL reading comprehension	0.147	0.034	<0.001
Grammar knowledge ➔ vocabulary	0.512	0.025	<0.001
Vocabulary ➔ EFL reading comprehension	0.651	0.027	<0.001
Indirect effect of grammar knowledge on EFL reading comprehension	0.333	0.021	<0.001

*Bootstrap sample size = 1,000*.

The testing of the GF mediation model revealed *χ*^2^ (7, *N* = 1,149) = 9.881, *p* < 0.05; *CFI* = 0.996; *TLI* = 0.991; and *RMSEA* = 0.036, 95% *CI* = 0.006–0.065, indicating a very good fit. The mediation models (Figure 3B) evaluated the strength of the indirect relationship while controlling for the direct effect of the GF subskill on EFL reading comprehension. In this model, vocabulary was indicated to mediate the relationship between the GF subskill and EFL reading comprehension.

As shown in Figure 3B, GF significantly predicted the vocabulary mediator (*β* = 0.388, *p* < 0.001), and the mediator vocabulary also significantly predicted EFL reading comprehension (*β* = 0.699, *p* < 0.001). GF and vocabulary combined explained about 53.2% of the variance within EFL reading comprehension. As listed in Table 6, GF (through vocabulary) also had a small but significant indirect effect on EFL reading comprehension (*β* = 0.271, *p* < 0.001). Both the direct and indirect effects were significant, as were the total effects of GF on EFL reading comprehension (*β* = 0.341, *p* < 0.001). The direct effect of GF predicted EFL reading comprehension after controlling for vocabulary and was significant and smaller than the total effect (*β* = 0.070, *p* < 0.05). However, the bootstrapped 95% CIs for both the total (0.236–0.306) and direct (0.023–0.117) effect models did not include zero, suggesting that there existed both direct and mediating effects of GF on EFL reading comprehension.

**Table 6:** Mediation effect of vocabulary in the GF model.

	*β*	SE	Value of *p*
GF subskill ➔ EFL reading comprehension	0.070	0.028	<0.05
GF subskill ➔ vocabulary	0.388	0.025	<0.001
Vocabulary ➔ EFL reading comprehension	0.699	0.022	<0.001
Indirect effect of GF subskill on EFL reading comprehension	0.271	0.020	<0.001

*Bootstrap sample size = 1,000*.

## Discussion

### Relative Roles of Vocabulary and Grammar Knowledge in EFL Reading Comprehension

Previous research findings regarding the relative contributions of vocabulary and grammar knowledge to EFL reading comprehension were inconclusive, which might be attributable to variations in the characteristics of the learners (e.g., language proficiency) and research designs (e.g., the definition and measurement of grammar knowledge; Choi and Zhang, 2021). Based on an adequate sample size of elementary students and comprehensive grammar knowledge, vocabulary size, and reading comprehension tests, our study indicated that both vocabulary and grammar knowledge played significant roles in EFL reading comprehension. However, vocabulary had a much more significant effect on the EFL reading of elementary students than did grammar knowledge. This finding also indicates that at the elementary-school stage, vocabulary influenced the EFL reading comprehension achievements more than grammar knowledge did. Our findings provide evidence that is consistent with that obtained in studies using a similar SEM approach, such as the investigations of Zhang (2012) (on graduate-level EFL learners) and Van Gelderen et al. (2004) (on middle school-level students) into the relative contribution of linguistic knowledge to L2 reading comprehension. However, our findings did not corroborate the findings of Shiotsu and Weir (2007), which indicated that L2 grammar knowledge had stronger predictive power than vocabulary for L2 reading comprehension achievement.

Notably, the stronger predictive effect of vocabulary for L2 reading comprehension still persisted after reducing the contextual influences of grammar knowledge tests. Previous researchers (Alderson, 1993; Urquhart and Weir, 1998) postulated that if a grammar knowledge test comprises items that require context to find the answer (e.g., the cloze test format) then the competence being tested would overlap the reading comprehension competence. This study tried to reduce the contextual effect of grammar knowledge by separating the meaning and form components, as proposed by Purpura (2004). The current study indicated that compared with using both meaning and form, using just the form component significantly reduced the correlation coefficients of grammar knowledge and vocabulary (from 0.82 to 0.39), and those of grammar knowledge and reading comprehension (from 0.55 to 0.24). However, despite the considerably lower correlation coefficients between form-based grammar knowledge and reading comprehension, the direct and indirect effects of grammar knowledge on reading comprehension remained, which indicated that grammar knowledge still exerted a significant and robust effect on the EFL reading comprehension of elementary-school students, although with a smaller effect than for vocabulary.

A possible reason for the findings that vocabulary had a more-significant influence on the reading comprehension of EFL elementary-school students than did grammar knowledge, may be that vocabulary was more critical for helping students identify contextual cues and deconstruct sentence and paragraph meanings in reading comprehension tasks. For EFL elementary-school learners, vocabularies (either in a format of pronounced sound or visualized letter sequence) are the most-accessible stimuli or material for learning English. Vocabularies may be seen as a kind of stepping stone to further the learning of English skills for beginners. Especially when reading an unfamiliar text in a reading test, EFL students tend to use their most-accessible tool to complete their objective of extracting meanings from each sentence, inferencing, and integrating the meanings among sentences, and finally, to form an overall meaning of the paragraph or text. Compared with vocabulary, the grammar knowledge of EFL students may play a minor role in reading tasks. Although previous researchers have postulated that grammar knowledge is important for building coherence, integrating information, and forming constructing text models in a reading task (Grabe, 2005; Zhang, 2012), these functions may only be exploited when learners reach a certain proficiency level of English (Choi and Zhang, 2021); when the proficiency does not reach a threshold, the function of grammar knowledge on reading comprehension may be not so obvious. Kim and Cho (2015) provided evidence for these speculations, indicating that for high school students with high English proficiency, grammar knowledge contributed more to EFL reading comprehension than did vocabulary; however, for high school students with intermediate proficiency, vocabulary had a more important role in EFL reading comprehension. For high-school students with low proficiency, both grammar knowledge and vocabulary had no significant predictive power for EFL reading comprehension. Within the Taiwan EFL elementary-school context, students only attend two or three classes each week, and it is difficult for them to apply certain sentence patterns within such a restricted learning time. Therefore, compared with the easier target of vocabularies, a relatively lower grammar knowledge proficiency may explain its lesser predictive power for the EFL reading comprehension of elementary-school students.

### Grammar Knowledge Exerted Its Influence on Reading Comprehension Through Vocabularies

In addition to comparing the relative direct effects of vocabularies and grammar knowledge on EFL reading comprehension, our study also indicated that grammar knowledge exerted both direct and indirect effects on reading comprehension, with the latter mediated by vocabulary. Moreover, whether or not grammar knowledge included meaning components, the mediation effects (*β* = 0.33 and 0.27 for with and without the meaning component, respectively) in the grammar knowledge-vocabulary-reading comprehension model were greater than the direct effects of grammar knowledge on reading comprehension (*β* = 0.147 and 0.070). The mediation effects of vocabulary provided more evidence for the roles of grammar knowledge and vocabulary on reading comprehension. Although the regression models indicated that grammar knowledge played a minor role in reading comprehension (a direct effect), the mediation model indicated that grammar knowledge not only directly influenced reading comprehension but also influenced the vocabularies followed by reading comprehension.

The finding that grammar knowledge predicted vocabulary knowledge corroborated previous research findings of syntactic knowledge augmenting vocabulary acquisition. For example, Naigles (1993) used the term “syntactic bootstrapping” to define the phenomena where children use syntactic structure knowledge derived from linguistic observations to determine word meanings. Similar findings have been obtained in L2 learning studies. For example, Paribakht (2004) and De Bot et al. (1997) indicated that when unfamiliar words were encountered in a reading task, lexical inference is one of the most-important strategies for learners to improve their reading comprehension. Nevertheless, the two types of knowledge most commonly employed as linguistic resources for making lexical inferences were sentence-level grammatical knowledge, which refers to knowledge about speech parts and syntactic relationships among words within a sentence (e.g., word order and word class), and word morphology knowledge, which refers to knowledge about grammatical inflections (e.g., “-s,” “-ed,” and “-ing”) and word derivations (e.g., stems and affixes). Ranjbar (2012) further indicated that grammar knowledge was an important factor in deciphering the meanings of unknown words, with more-comprehensive grammar knowledge increasing the proficiency level of learners in guessing words. Zhang and Koda (2012) employed SEM to investigate the relationships of morphological awareness, vocabulary knowledge, and reading comprehension, and found that the morphological awareness of adult EFL learners both directly influenced reading comprehension and indirectly influenced reading comprehension with vocabulary as a mediator. The above findings support our research findings for the grammar knowledge of EFL learners possibly play roles of scaffolding and hinting which are helpful for vocabulary learning and facilitating reading comprehension.

## Conclusions and Implications

There were three main findings in the current study. First, by analyzing EFL elementary-school students who were beginners in learning English, we have provided evidence that vocabularies play a more-significant role in EFL reading comprehension than does grammar knowledge for EFL beginners. This evidence fills the gap in previous research that had focused on adult or teenager EFL learners. Second, by using grammar knowledge tests composed of both GM and GF, we were able to provide a clearer picture of the relationships among vocabulary, grammar knowledge, and reading comprehension. This study has indicated that even if the possible contaminating effect of GM is removed, grammar knowledge still plays a minor but significant role in EFL reading comprehension. Third, the SEM regression and mediation models indicated that in addition to the direct effect of grammar knowledge on reading comprehension, grammar knowledge also exerts an indirect effect on reading comprehension, with vocabulary as a mediator. This finding expands previous research focused on comparing the relative effects of grammar knowledge and vocabulary on reading comprehension.

The above findings have at least two possible implications for future EFL teaching practices and research. First, given that grammar can mostly help comprehension through its influence on vocabulary, elementary-school EFL pedagogy/curriculum designers who value grammar instruction can consider integrating grammar knowledge with scaffolding vocabulary teaching methods in the class. Instead of explaining grammar rules with difficult terminology, the inductive instructional methods, such as a communicative grammar teaching approach (e.g., Underwood, 2017) or consciousness-raising tasks (e.g., Fotos, 1994; Takimoto, 2006) in which grammatical rules are not presented or explained explicitly to learners (VanPatten and Oikkenon, 1996; Benitez-Correa et al., 2019) may be more appropriate for the EFL elementary class. To familiarize students with the sentence patterns, teachers may consider utilizing visual and audio input-enhancement techniques (Doughty and Williams, 1998) to elicit students’ consciousness of noticing linguistic features and word order, and further, help them construct sentence patterns through communication practice. Second, EFL researchers should consider investigating the interaction of vocabulary and grammar in depth, particularly the possible mutual enhancement or trade-off between these two components. Although our findings illustrate a possible interaction between vocabulary and grammar knowledge on reading comprehension for young EFL learners, further investigation of the interacting effects of these two components in learners with different learning stages and proficiency levels will yield more-meaningful evidence on the mechanisms of foreign language learning for EFL students.

## Data Availability Statement

The original contributions presented in the study are included in the article/Supplementary Material, further inquiries can be directed to the corresponding author.

## Ethics Statement

Ethical review and approval was not required during the period of data collection (2016-2017) for the study on human participants in accordance with the local legislation and institutional requirements. Written informed consent to participate in this study was provided by the participants’ legal guardian/next of kin.

## Author Contributions

T-CH and Y-TS conceived and designed the study, collected and analyzed the data, and supervised the data analysis. T-JC and Y-TC assisted with data analyses. T-CH, Y-TS, and H-HL wrote the first draft of the manuscript. All authors contributed to the article and approved the submitted version.

## Funding

Parts of the data collection were supported by the Ministry of Science and Technology, Taiwan (MOST 104-2511-S-003-012-MY3); parts of the data analyses were supported by the Ministry of Science and Technology (MOST 111-2622-8-003-001–TH, 110-2511-H-003-033-MY3, and 110-2511-H-003-051) and the Higher Education Sprout Project of the Ministry of Education, Taiwan.

## Conflict of Interest

The authors declare that the research was conducted in the absence of any commercial or financial relationships that could be construed as a potential conflict of interest.

## Publisher’s Note

All claims expressed in this article are solely those of the authors and do not necessarily represent those of their affiliated organizations, or those of the publisher, the editors and the reviewers. Any product that may be evaluated in this article, or claim that may be made by its manufacturer, is not guaranteed or endorsed by the publisher.
