# Supplementary material for: Relative Roles of Grammar Knowledge and Vocabulary in the Reading Comprehension of EFL Elementary-School Learners: Direct, Mediating, and Form/Meaning-Distinct Effects
Source: Front Psychol. 2022 Jun 21;13:827007. doi: 10.3389/fpsyg.2022.827007 (PMC9255428; doi:10.3389/fpsyg.2022.827007)
Supplement: Supplementary file 1 [file Data_Sheet_1.PDF]

APPENDIX A. Examples of DCEC-RC items

DCEC 2: Information extraction (The test-taker will need to identify “summer” and “red bar” and first determine what the red bar is by reading the text, and then get the best answer, which is (B) Happy Zoo.)

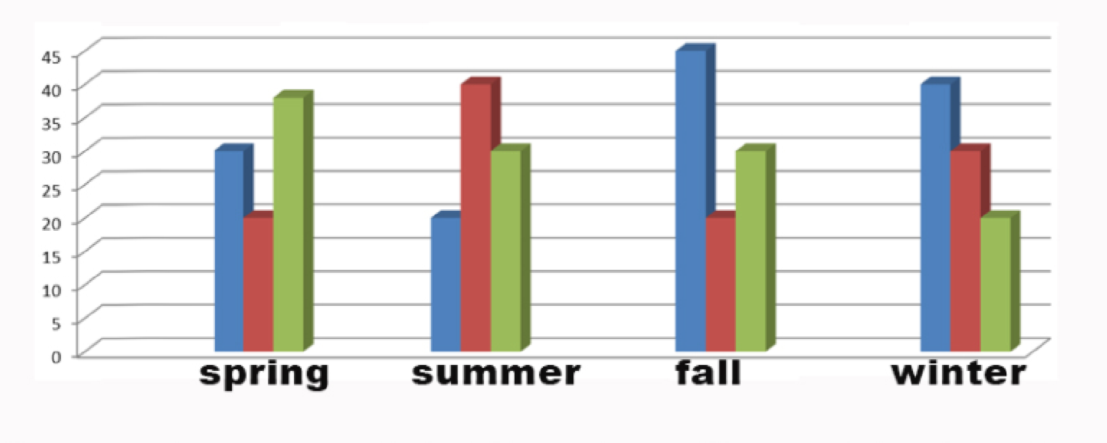

There are three zoos: Taipei Zoo, Happy Zoo, and Joy Zoo. Do you know how many students go there in spring, summer, fall, and winter? Please look at the bar chart here. The blue bar is Taipei Zoo, the red bar is Happy Zoo, and the green bar is Joy Zoo.

Which zoo has the most visitors in summer?

(A) Taipei Zoo.

(B) Happy Zoo.

(C) Joy Zoo.

DCEC 2: Information integration (The test-taker will need to identify key words such as “fat,” “red,” and “big red bag,” and integrate all the information to match the characteristics of the men shown in the pictures to get the correct answer.)

I am fat, I am red,
And my home is far away.
I have many gifts in my big red bag.

Who am I? You can’t see.
Just be nice and go to sleep,
You will have a gift under the tree.

Who am I?

A.

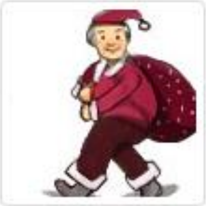

B.

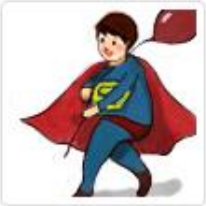

C.

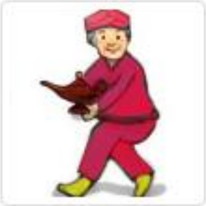

DCEC 2: Inference (The test-taker will need to identify the key sentences of "Just be nice and go to sleep. You will have a gift under the tree," and then infer the possible reason why Little John doesn't get a gift in order to obtain the correct answer.)

I am fat, I am red,
And my home is far away.
I have many gifts in my big red bag.

Who am I? You can't see.
Just be nice and go to sleep,
You will have a gift under the tree.

Little John doesn't get a gift. Which is the most likely reason?
(A) He is fat and red.
(B) He doesn't go to bed at night.
(C) He has a tree at home.
